# An expert review on the use of tenofovir alafenamide for the treatment of chronic hepatitis B virus infection in Asia

**DOI:** 10.1007/s00535-020-01698-4

**Published:** 2020-07-14

**Authors:** Michael R. Charlton, Altaf Alam, Akash Shukla, Bekhbold Dashtseren, Cosmas Rinaldi Adithya Lesmana, Davadoorj Duger, Diana Alcantara Payawal, Do Duy Cuong, Ganbolor Jargalsaikhan, Ian Homer Yee Cua, Jose Decena Sollano, Karam Romeo Singh, Kaushal Madan, Khin Maung Win, Khin Pyone Kyi, Kyaw Soe Tun, Mohd. Salih, Mukul Rastogi, Neeraj Saraf, Pham Thi Thu Thuy, Pham Tran Dieu Hien, Rino Alvani Gani, Rosmawati Mohamed, Tawesak Tanwandee, Teerha Piratvisuth, Wattana Sukeepaisarnjaroen, Win Naing, Zahid Yasin Hashmi

**Affiliations:** 1 Transplant Institute, Center for Liver Diseases, University of Chicago Biological Sciences, 5841 South Maryland Avenue, Chicago, Illinois USA; 2 GI Hepatology, Lahore, Pakistan; 3 Department of Gastroenterology, LTM Medical College and Sion Hospital, Maharashtra Mumbai, India; 4 Liver Center Hospital, Ulaanbaatar, Mongolia; 5 Department of Internal Medicine, Hepatobiliary Division, Dr. Cipto Mangunkusumo Hospital, Universitas Indonesia, Jakarta, Indonesia; 6 Mongolian National University of Medical Sciences, Ulaanbaatar, Mongolia; 7 Department of Medicine, Cardinal Santos Medical Center, Mandaluyong, Metro, Manila, Philippines; 8 Department of Infectious Diseases and HIV Outpatient Clinic, Bach Mai Hospital, Hanoi, Vietnam; 9 Department Liver Center, Ulaanbaatar, Mongolia; 10 Department International Graduate Program in Medicine (IGPM) Institution, Ulaanbaatar, Mongolia; 11 College of Medicine, Taipei Medical University, Taipei, Taiwan; 12 Research Committee and Social Committee, Institute of Digestive and Liver Diseases, St. Luke’s Medical Center, Taguig, Philippines; 13 Department of Medicine, University of Santo Tomas Hospital, Manila, Philippines; 14 Liver Clinic, Regional Institute of Sciences, Imphal, India; 15 Gastroenterology & Hepatology, Max Smart Super Speciality Hospital, Saket, New Delhi, India; 16 University of Medicine (1) YGN, Yangon, Myanmar; 17 Myanmar Liver Foundation, Liver Foundation, Yangon, Myanmar; 18 Myanmar GI and Liver Society, Yangon, Myanmar; 19 Department of Hepatology, Quaid e Azam International Hospital, Islamabad, Pakistan; 20 Department of Hepatology and Gastroenterology, Fortis Hospital, Noida, India; 21 Department of Transplant Hepatology, Fortis Hospital, Noida, India; 22 Clinical/Transplant Hepatology Institute of Digestive and Hepatobiliary Sciences Medanta, The Medicity, Gurgaon, New Delhi India; 23 Department of Medic Medical Center, Ho Chi Minh City, Vietnam; 24 Department of Infectious Disease, Pham Ngoc Thach University of Medicine, Ho Chi Minh, Vietnam; 25 Liver Transplantation team, Ciptomangunkusumo Hospital, Jakarta, Indonesia; 26 Department of Medicine, University Malaya Medical Centre, Kuala Lumpur, Malaysia; 27 Division of Gastroenterology, Department of Medicine, Faculty of Medicine, Siriraj Hospital, Mahidol University, Bangkok, Thailand; 28 Department of Medicine, NKC Institute of Gastroenterology and Hepatology, Songklanagarind Hospital, Prince of Songkla University, Hat Yai, Thailand; 29 Gastroenterology Unit, Department of Medicine, Srinagarind Hospital, Faculty of Medicine, Khon Kaen University, Khon Kaen, Thailand; 30 Department of Hepatology, Yangon General Hospital, University of Medicine (1), Yangon, Myanmar; 31 PMC, Allied and DHQ Hospital, Faisalabad, Pakistan

**Keywords:** Hepatitis B virus, Nucleoside analogs, Tenofovir alafenamide, Asia

## Abstract

Asia has intermediate-to-high prevalence and high morbidity of hepatitis B virus (HBV) infection. The use of guideline-recommended nucleos(t)ide analogs with high barrier to resistance, such as entecavir (ETV), tenofovir disoproxil fumarate (TDF), and tenofovir alafenamide (TAF), is one of the key interventions for curbing HBV infection and associated morbidity in Asia. However, there are some challenges to the use of ETV and TDF; while ETV is associated with high resistance in lamivudine (LAM)-exposed (especially LAM-refractory) patients; bone and renal safety issues are a major concern with TDF. Hence, a panel of twenty-eight expert hepatologists from Asia convened, reviewed the literature, and developed the current expert opinion-based review article for the use of TAF in the resource-constrained settings in Asia. This article provides a comprehensive review of two large, phase 3, double-blind, randomized controlled trials of TAF versus TDF in HBeAg-negative (study 0108) and HBeAg-positive (study 0110) chronic HBV patients (> 70% Asians). These studies revealed as follows: (1) non-inferiority for the proportion of patients who had HBV DNA < 29 IU/mL; (2) significantly high rate of normalization of alanine aminotransferase levels; (3) no incidence of resistance; and (4) significantly better bone and renal safety, with TAF vs. TDF up to 144 weeks. Considering the benefits of TAF, the expert panel proposed recommendations for optimizing the use of TAF in Asia, along with guidance on specific patient groups at risk of renal or bone disease suitable for TAF therapy. The guidance provided in this article may help clinicians optimize the use of TAF in Asia.

## Introduction

### Prevalence and burden of chronic hepatitis B virus infection in Asia

Globally, chronic hepatitis B virus (HBV) infection was prevalent in 257 million individuals (3.5%) in 2015. Asia comprises more than 40 countries from the Western Pacific, Southeast Asia, and Eastern Mediterranean regions; these regions were reported to have the first, third, and fourth highest prevalence of chronic HBV infection in 2015, accounting for about 115, 39 and 21 million HBV-infected individuals, respectively [1]. The endemicity of HBV within Asia is heterogeneous, with most of the regions having an intermediate-to-high prevalence of HBV infection [2–9].

Not only is the prevalence of HBV infection high in Asia but also is the morbidity and mortality from HBV infection. Single-center studies conducted across various regions in Asia report that HBV infection is the leading cause of cirrhosis [10, 11]. A systematic review by Ashtari et al*.*, of all published studies before 2014, on hepatocellular carcinoma (HCC) in Asia, revealed that over 70% of all global new liver cancer cases were diagnosed in Asia and that chronic HBV was the main cause of HCC in this region [12]. In another recent study to understand the global patterns of HCC risk by geographic region, it was noted that majority of the global liver cancer cases attributable to HBV infection occurred in Asia [13]. Asia also bears the highest burden of mortality from viral hepatitis; globally, the highest mortality rates have been reported in the Western Pacific region (24.1 deaths per 100, 000), followed by the Southeast Asia region (21.2 per 100, 000) [1].

Some of the plausible reasons for the high prevalence and morbidity of chronic HBV infection in Asia are as follows (1) the high rates of mother-to-child disease transmission; (2) very low rates of antiviral treatment in pregnant women with high viral load; and (3) very low rates of uptake of birth dose vaccination and hepatitis B immunoglobulin G (HBIG) [1, 14] In Asia, the risk of mother-to-child transmission of HBV infection in hepatitis B surface antigen (HBsAg)-positive and hepatitis B e-antigen (HBeAg)-positive mothers is 70–100%, and HBsAg-positive and HBeAg-negative mothers is 5–30% [1] The percentage of HBV-infected pregnant women treated with antiviral therapy in 2016 has been estimated to be < 1%, 0%, and 1% in the Western Pacific, Southeast Asian, and Eastern Mediterranean regions, respectively. While the proportion of infants with timely uptake of birth dose HBV vaccination in the Western Pacific, Southeast Asian, and Eastern Mediterranean regions in 2016 has been reported to be 88%, 47%, and 26%, respectively, the corresponding rates for the proportion of infants (of HBsAg-positive mothers who received HBIG) with uptake of HBIG and full vaccination has been noted to be 54%, 2%, and 7%, respectively, in 2016 [14].

The intermediate-to-high prevalence and high morbidity estimates, along with high rates of mother-to-child disease transmission and low HBV vaccination rates, reflect upon an urgent need for optimization of treatment of HBV infection in Asia [1, 3].

### Treatment of chronic HBV infection: current guideline recommendations

Currently, two main strategies are available for the treatment of chronic HBV infection: Pegylated interferon-α (PegIFNα) and nucleos(t)ide analogs (NAs) [15, 16]. The high variability of response and unfavorable safety profile limit the use of PegIFNα [16]. Treatment of chronic HBV-infected patients with NAs may be a preferred option to PegIFNα to achieve safe, sustained, and potent antiviral suppression [15, 17]. Among NAs, while lamivudine (LAM), adefovir dipivoxil (ADV), and telbivudine (TBV) represent a class of NAs with a low barrier against HBV resistance, entecavir (ETV), tenofovir disoproxil fumarate (TDF), and tenofovir alafenamide (TAF) may be classified as NAs with a high barrier against HBV resistance [16].

The 2017 guidelines from the European Association for the Study of the Liver (EASL) recommend ETV, TDF, or TAF as the preferred first-line monotherapy options for the management of all adults with chronic HBV infection. Additionally, EASL recommends the following guidelines for treatment for HBV infection: [16]

- Treatment with antiviral agents should be initiated in all chronic HBV-infected patients (HBeAg positive or negative), defined by HBV DNA > 2000 IU/mL, ALT > upper limit of normal (ULN) and/or at least moderate liver necroinflammation or fibrosis.
- Treatment should be initiated in all patients with compensated or decompensated cirrhosis, irrespective of ALT levels or any detectable HBV DNA. Patients with decompensated cirrhosis should be treated with NAs with high barrier to resistance.
- Patients with HBV DNA > 20,000 IU/mL and ALT > 2xULN should be initiated on HBV antiviral therapy, regardless of the degree of fibrosis.
- Patients with HBeAg-positive chronic HBV infection, defined by persistently normal ALT and high HBV DNA levels, may be treated if they are older than 30 years, regardless of the severity of liver histological lesions.
- Patients with HBeAg-positive or -negative chronic HBV infection and family history of HCC or cirrhosis and extrahepatic manifestations can be treated, even if typical treatment indications are not fulfilled.
- Patients with HBV-related liver disease, and on the waiting list for liver transplant, should be treated with an NA.
- Combination of HBIG and a potent NA is recommended for the prevention of HBV recurrence post-liver transplantation; patients with low risk of recurrence may discontinue HBIG but should be on continuous prophylaxis with NAs.

The American Association for the Study of Liver Diseases (AASLD) 2018 guidelines recommend ETV, TDF, or TAF as the preferred first-line therapies for the management of adults with immune-active, chronic HBV infection. The guidelines also recommend ETV or TAF over TDF in patients with or at risk of bone or renal disease. Further, for the management of immune-tolerant, chronic HBV-infected patients, AASLD guidelines recommend antiviral therapy only in adults aged > 40 years, with (1) normal alanine aminotransferase (ALT) levels, but elevated HBV DNA (1,000,000 IU/mL), and (2) liver complications such as necroinflammation or fibrosis. In adult patients with compensated cirrhosis, the guidelines recommend antiviral therapy, preferably with ETV, TDF or TAF, regardless of ALT level or level of viremia, to reduce the risk of decompensation. In HBsAg-positive adults with decompensated cirrhosis, indefinite antiviral therapy with ETV or TDF is recommended, regardless of HBV DNA, HBeAg, or ALT level, to decrease the risk of worsening liver-related complications; TAF or ETV may be considered in cases with renal dysfunction and/or bone disease. In all HBsAg-positive patients undergoing liver transplantation, AASLD recommends prophylaxis with ETV, TDF, or TAF with or without HBIG, regardless of HBeAg status. A long-term antiviral therapy, preferably with ETV, TDF, or TAF, is recommended in all HBsAg-negative patients receiving HBsAg-negative, but anti-hepatitis B core antigen (HBc)-positive grafts [15].

The Asian Pacific Association for the Study of the Liver (APASL) has updated the recommendations for the management of HBV infection in 2015 and, hence, has not incorporated TAF for the treatment of chronic HBV infection into the guidelines. According to APASL, TDF and ETV are the preferred NAs for the first-line therapy of chronic HBV infection, including patients with compensated or decompensated cirrhosis [18]. However, according to the latest Taiwan consensus statement, ETV, TDF, and TAF are potent HBV inhibitors with a high barrier to resistance and should be preferably used as first-line monotherapies for the treatment of chronic HBV infection [19]. The latest expert consensus for the management of chronic HBV infection in Asian Americans also recommend ETV, TDF, or TAF as the preferred first-line options [20]. The 2018 Indian National Association for Study of the Liver (INASL) guidelines also strongly recommend lifelong antiviral therapy with NAs with high barrier to resistance, such as ETV, TDF, or TAF, in patients with decompensated cirrhosis, including those undergoing liver transplantation, irrespective of HBV replication [21].

### Treatment of chronic HBV infection with nucleos(t)ide analogs in Asia: current challenges

All three NAs with high barrier against HBV resistance, ETV, TDF, and TAF, recommended by the key guidelines, have been clinically proven to be effective and safe for the treatment of chronic HBV infection and HBV-related cirrhosis. A systematic review and meta-analysis of 20 studies comparing the efficacy of ETV versus TDF in chronic HBV patients reported a significant difference in viral suppression and improvement of liver function, in favor of TDF in the short term at 3 months. This difference, however, did not persist in the long term and in patients with HBV-related cirrhosis [22]. Both ETV and TDF have been clinically proven to be effective in the long term for the treatment of patients with chronic HBV infection, including those who relapsed after PegIFNα therapy [23–26]. Further, ETV has also been found to be effective in patients with chronic kidney disease, including those on hemodialysis, without affecting the renal function [27]. There is also an abundance of literature supporting the efficacy of safety of TAF for the treatment of HBV-infected patients, which will be discussed in detail, in the following sections.

Despite the availability of effective NAs, such as ETV, TDF, or TAF, most countries in the Asia–Pacific region use NAs with a low barrier against HBV resistance, as the first-line treatment options for the management of chronic HBV infection, due to lack of awareness of the availability of effective treatments, cost/access issues, and lack of adequate reimbursement policies for treatment [18, 28–30]. The use of NAs with low barrier to HBV resistance may in turn result in the emergence of multi-drug-resistant HBV variants with limited salvage options [18]. Further, even among NAs with a high barrier against HBV resistance, ETV and TDF have several shortcomings, as described below.

#### Limitations of Entecavir

Paradoxically, while only 1–2% resistance against HBV is noted in NA-naïve patients treated with over 5 years of ETV monotherapy [31, 32], high incidence of ETV resistance has been reported in patients resistant to LAM therapy. In LAM-refractory patients, the probability of ETV resistance through 5 years of treatment has been noted to be as high as 43% and 51%, with and without virological breakthrough, respectively [32].

Resistance to long-term LAM monotherapy develops due to the substitution of methionine with valine or isoleucine in the tyrosine–methionine–aspartate–aspartate (YMDD) motif at amino acid position 204 in the C domain of the HBV DNA polymerase enzyme gene, leading to the development of rtM204V/I mutations, which are usually associated with other mutations, such as rtL180M, rtV173L, and rtL80V/I. These mutations lead to an increase in HBV replication [33]. The incidence of resistance mutations has been reported to be about 70% after 5 years of LAM therapy [34]. Lamivudine resistance may also develop in LAM-untreated patients due to spontaneous structural changes in the HBV DNA polymerase enzyme [35, 36]. In a systematic review and meta-analysis to assess the overall incidence and related risk factors of YMDD motif mutations among LAM-naïve chronic HBV carriers from eight countries across Asia and Europe, the pooled incidence of these mutations was found to be 12.21% [36]

In addition to LAM-refractory patients, ETV resistance has also been found to develop in LAM-exposed patients, with no prior LAM resistance, thus highlighting the need for close monitoring of patients treated with ETV, irrespective of the presence or absence of LAM resistance [31].

Another shortcoming with ETV therapy is the emergence of human immunodeficiency virus (HIV)-resistant strains when the drug is used for the treatment of HBV infection in patients with HBV/HIV co-infection. This finding highlights the need for caution when using ETV in HBV/HIV co-infected patients not receiving suppressive antiretroviral therapies [37, 38].

#### Limitations of TDF

Despite potent antiviral activity and no resistance with long-term use [39], bone and renal safety issues have been a major concern with the use of TDF [40–44], due to the high systemic exposure of its active metabolite, tenofovir diphosphate (TFV-DP) [45]. A significant and independent association has been reported between the use of TDF and altered excretion of retinol-binding protein (RBP)/creatinine, and subclinical tubular damage in chronic HBV-infected patients [46]. This risk of proximal tubular dysfunction has been noted to significantly increase with long-term (≥ 18 months) TDF therapy [47]. The degree of renal impairment with TDF in chronic hepatitis B-infected patients with preserved renal function at baseline, in a recent real-world setting in Asia, has been noted to be about 2.9%, 1.8%, and 1.7% after 1, 2, and 3 years of treatment, respectively [48]. In another real-world setting in Asia, about 10.9% of chronic HBV-infected patients were noted to develop renal insufficiency with up to 96 weeks of TDF therapy [49]. Hence, close monitoring of RBP and markers of renal tubular damage and dysfunction needs to be done to help early detection of TDF-related renal toxicity.

## Rationale and methodology for development of the current expert opinion-based review

Considering the challenges pertaining to the high prevalence, morbidity and mortality of HBV infection, and low rates of treatment with antiviral agents in Asia, along with limitations of ETV and TDF and advantages of TAF over other antiviral agents, a panel of 28 (twenty eight) expert hepatologists from Asia convened, reviewed the literature, and developed the current expert opinion-based review article for the use of TAF in the resource-constrained settings in Asia. The recommendations proposed in this article are based on the existing clinical evidences and the expert opinion shared by the panel.

## Tenofovir alafenamide: a safer alternative to TDF

### Pharmacology of TAF

Tenofovir alafenamide is a phosphonamidate prodrug of tenofovir. After oral absorption, it is hydrolyzed in the hepatocytes to tenofovir, which is then phosphorylated to the active metabolite, TFV-DP. Incorporation of TFV-DP into the HBV DNA, results in termination of HBV replication [50–52].

The recommended dosage of TAF is 25 mg once daily, to be taken orally with food. No dosage adjustment is required in patients with estimated creatinine clearance (CrCl) ≥ 15 mL/min, in patients with CrCl < 15 mL/min who are receiving hemodialysis or in those with hepatic impairment. There are no dosing recommendations for TAF in patients with CrCl < 15 mL/min who are not receiving hemodialysis [53, 54]. The use of TAF may be considered in pregnancy, only if necessary, as there is limited or no data on the use of TAF in this population. The use of TAF is not recommended during breast feeding [54].

Tenofovir alafenamide is transported by P-glycoprotein (P-gp) and breast cancer resistance protein (BRCP). Co-administration of TAF with inhibitors of P-gp and BRCP may increase the plasma concentration of TAF, and concomitant administration of TAF with P-gp inducers may decrease the plasma concentrations of TAF, resulting in loss of therapeutic activity. Therefore, administration of TAF with inhibitors of P-gp or BRCP, or P-gp inducers should be avoided [53, 54].

Although both TAF and TDF are prodrugs of tenofovir and are metabolized to TFV-DP [53, 54], TAF results in more than 90% lower systemic exposure of tenofovir and provides higher intracellular levels of TFV-DP to target cells than TDF at therapeutic doses [55, 56]. Hence, TAF has fewer renal and bone safety issues compared to TDF.

### Clinical Efficacy of TAF

Evidence for the clinical efficacy of TAF comes from (1) two large, phase 3 clinical trials (study 0108 in HBeAg-negative, and study 0110 in HBeAg-positive chronic HBV patients) with endpoints evaluated at 48 weeks [57, 58]; (2) pooled analysis of studies 108 and 110 conducted at 96 and 144 weeks [43, 59]; and (3) open-label extension phase of studies 108 and 110 (144 weeks of TAF therapy) [60, 61]. A majority of patients in both 108 and 110 studies belonged to the Asian ethnicity. Apart from these two studies, few other real-world studies conducted in Asia have also evaluated the efficacy and safety of TAF for the management of HBV infection.

#### Design and endpoints in studies 0108 and 0110

Studies 0108 and 0110 were randomized, double-blind, multinational, non-inferiority studies. Inclusion criteria were treatment-naïve and treatment-experienced patients aged ≥ 18 years with chronic HBV infection with plasma HBV DNA > 20,000 IU/mL, serum ALT > 60 U/L in men or > 38 U/L in women (and not more than ten times ULN), and estimated creatinine clearance of at least 50 mL/min. Randomization in both the studies was done in a 2:1 ratio in a double-blind manner to TAF 25 mg or TDF 300 mg orally, once daily, each with a matching placebo for up to 96 weeks [57, 58]. After 96 weeks, following a protocol amendment, the double-blind phase was extended for an additional year in about half of the patients and the remaining half received open-label therapy with TAF 25 mg orally once daily until week 144 [59].

Both the studies had the same primary efficacy endpoint, viz. the proportion of patients who had HBV DNA < 29 IU/mL at week 48. Other pre-specified efficacy endpoints included the following: (1) percentage of patients with ALT normalization at week 48 (ALT > ULN of ≤ 43 U/L for men and ≤ 34 U/L for women younger than 69 years of age; ≤ 35 U/L for men and ≤ 32 U/L for women older than 69 years of age; at no more than ten times the ULN by central laboratory normal range; or 30 U/L for men and 19 U/L for women as per AASLD); and (2) proportion of patients with HBsAg loss and seroconversion to anti-HBs at week 48. Bone and renal safety were the secondary safety endpoints [57, 58].

#### Efficacy of TAF through 144 weeks in studies 108 and 110

While 426 eligible HBeAg-negative chronic HBV patients were randomized to TAF (*n* = 285) or TDF (*n* = 141) at 105 sites in study 108, study 110 was conducted at 161 centers across 19 countries in 873 eligible HBeAg-positive chronic HBV patients who were randomized to TAF (*n* = 581) or TDF (*n* = 292) [57, 58]. The pooled analysis of both the studies at 96 weeks included 866 patients on TAF and 432 patients on TDF [43]. About 1118 patients were included in the analysis of the double-blind extension phase [759 HBeAg-positive and 359 HBeAg-negative chronic HBV patients; 866 in the TAF group (both double-blind extension and open-label phases), and 252 in the TDF group (double-blind extension phase)] [59]. About 72% in each group in study 108, 83% in the TAF group and 79% in the TDF group in study 110, 79% in the TAF group and 77% in the TDF group in the pooled analysis at 96 weeks, and 78% in the double-blind extension phase of both the studies were Asians [43, 57–59].

Treatment with TAF was found to be non-inferior to TDF at 48, 96, and 144 weeks in both HBeAg-negative and HBeAg-positive chronic HBV-infected patients (Tables 1, 2). Further, a pre-defined subgroup analysis revealed no significant difference in the proportion of patients achieving the primary endpoint between Asians and non-Asians in both the studies at both 48 and 96 weeks. Normalization of ALT levels was found to be significantly high in patients treated with TAF vs. TDF at week 96, by central laboratory criteria and AASLD criteria, and at week 144 by AASLD criteria in both the studies (Tables 1, 2) [43, 57–59]. There was no significant difference between the two groups in terms of HBeAg or HBsAg seroconversion or loss, at weeks 48, 96, and 144 in both the studies [43, 57–59].

**Table 1:** Efficacy of TAF vs. TDF at 48, 96 and 144 weeks in HBeAg-negative, chronic HBV patients [43, 57, 59]

Assessment parameter	Percentage of patients at 48 weeks				Percentage of patients at 96 weeks				Percentage of patients at 144 weeks*		
	TAF (%)	TDF (%)	Difference in proportions (95% CI)	*p* Value	TAF (%)	TDF (%)	Difference in proportions (95% CI)	*p* Value	TAF	TDF	*p* Value
HBV DNA < 29 IU/mL	94	93	1.8% (–3.6 to 7.2)	0.47	90	91	–0.6% (–7.0% to 5.8%)	0.84	87%	85%	NS
Normalized ALT by central laboratory criteria	83	75	8.0% (–1.3 to 17.2)	0.076	81	71	9.8% (0.2% to 19.3%)	0.038	NA	NA	NA
Normalized ALT by AASLD criteria	50	32	17.9% (8.0 to 27.7)	0.0005	50	40	10.9% (0.8% to 21.0%)	0.035	71%	59%	0.052

*TAF* tenofovir alafenamide, *TDF* tenofovir disoproxil fumarate, *HBeAg* hepatitis B e-antigen, *HBV* hepatitis B virus, *CI* confidence interval, *DNA* deoxy ribonucleic acid, *ALT* alanine aminotransferase; *AASLD* American Association for the Study of Liver Diseases, *NS* non-significant, *NA* not available

*Double-blind extension phase through 3 years

**Table 2:** Efficacy of TAF vs. TDF at 48, 96 and 144 weeks in HBeAg-positive, chronic HBV patients [43, 58, 59]

Assessment parameter	Percentage of patients at 48 weeks				Percentage of patients at 96 weeks				Percentage of patients at 144 weeks*		
	TAF (%)	TDF (%)	Difference in proportions (95% CI)	*p* Value	TAF (%)	TDF (%)	Difference in proportions (95% CI)	*p* Value	TAF	TDF	*p* Value
HBV DNA < 29 IU/mL	64	67	–3.6% (–9.8 to 2.6%)	0.25	73	75	–2.2% (–8.3 to 3.9%)	0.47	74%	71%	NS
Normalized ALT by central laboratory criteria	72	67	4.6% (–2.3 to 11.4%)	0.18	75	68	8.0% (1.2 to 14.7%)	0.017	NA	NA	NA
Normalized ALT by AASLD criteria	45	36	8.7% (1.8% to 1.6%)	0.014	52	42	10.6% (3.6 to 17.6%)	0.003	64%	53%	0.010

*TAF* tenofovir alafenamide, *TDF* tenofovir disoproxil fumarate, *HBeAg* hepatitis B e-antigen, *HBV* hepatitis B virus, *CI* confidence interval, *DNA* deoxy ribonucleic acid, *ALT* alanine aminotransferase, *AASLD* American Association for the Study of Liver Diseases, *NS* non-significant, *NA* not available

*Double-blind extension phase through 3 years

#### Efficacy of TAF in real-world settings in Asia

Studies evaluating the efficacy and safety of TAF in real-world settings in Asia are limited. A recent study by Kaneko et al*.* compared the efficacy and safety of TAF (*n* = 67) versus TDF (*n* = 117) and investigated the efficacy of switch-over from TDF to TAF therapy (*n* = 36). The percentage of patients with ALT normalization was numerically higher in the TAF versus TDF group at week 48 of treatment by both hospital-based criteria (100% vs. 83.3%, respectively) and AASLD laboratory criteria (57.1% and 45.2%, respectively). The decline in HBV DNA and HBsAg levels was comparable between both the groups at week 48 [62].

Furthermore, in a recent systematic review and network meta-analysis of 42 randomized controlled trials, while both TAF and TDF were found to be the best antiviral agents for virologic response, TAF was noted to be the best for ALT normalization among the assessed antiviral agents for the treatment of chronic HBV infection in both HBeAg-positive and HBeAg-negative patient population [63].

### Resistance with TAF

In the pooled analysis of studies 108 and 110 at 96 weeks, overall, no resistant isolates were detected in the TAF group [43, 64]. In the phenotypic analysis at week 144 of the double-blind extension phase in 49 patients, no isolates showed resistance to TAF [65]. Further, in vitro studies have revealed potent antiviral activity of TAF against LAM-, ETV-, and ADV-resistant isolates when compared to the wild-type HBV clinical isolates [66].

### Safety and tolerability with TAF

#### Common adverse events

Tenofovir alafenamide was well tolerated in studies 108 and 110, with upper respiratory tract infection, headache, and nasopharyngitis, being the most common adverse events [57, 58].

#### Bone safety (up to 144 weeks)

The decline in bone mineral density (BMD) at the hip and spine was significantly low with TAF vs. TDF at 48, 96 and 144 weeks in both the studies (Table 3) [43, 57–59]. Further, the magnitude of the difference in BMD decrease at the hip between the TAF and TDF groups was significantly greater at 96 weeks when compared to the difference observed at 48 weeks (*p* < 0.001); however, this effect was not noted at the spine [43].

**Table 3:** Change in hip and spine BMD from baseline with TAF vs. TDF at 48, 96 and 144 weeks in HBeAg-negative and HBeAg-positive chronic HBV patients [43, 57–59]

Assessment parameter	At 48 weeks (HBeAg-negative patients)				At 48 weeks (HBeAg-positive patients)				At 96 weeks in the pooled analysis*			At 144 weeks in the pooled analysis*		
	TAF (%)	TDF (%)	Adjusted % difference (95% CI)	*p* Value	TAF (%)	TDF (%)	Adjusted % difference (95% CI)	*p* Value	TAF (%)	TDF (%)	*p* Value	TAF (%)	TDF (%)	*p* Value
Mean % change in hip BMD from baseline	–0.29	–2.16	1.87% (1.42–2.32)	< 0.0001	–0.10	–1.72	1.62% (1.27–1.96)	< 0.0001	–0.33	–2.51	< 0.001	–0.4	–2.5	< 0.001
Mean % change in spine BMD from baseline	–0.88	–2.51	1.64% (1.01–2.27)	< 0.0001	–0.42	–2.29	1.88% (1.44–2.31)	< 0.0001	–0.75	–2.57	< 0.001	–0.5	–2.0	< 0.001

*TAF* tenofovir alafenamide, *TDF* tenofovir disoproxil fumarate, *HBeAg* hepatitis B e-antigen, *HBV* hepatitis B virus, *CI* confidence interval, *BMD* bone mineral density

*Both HBeAg-positive and HBeAg-negative patients

An exploratory analysis in study 108 revealed that the proportion of HBeAg-negative HBV patients experiencing > 3% reduction in BMD at the hip and spine was significantly less in the TAF vs. TDF group at week 48 (hip: 10% vs. 33%, *p* < 0.0001; spine: 22% vs. 39%, *p* = 0.0004, respectively) [57]. Further, pooled analysis at week 96 in both HBeAg-negative and HBeAg-positive patients revealed that fewer patients in the TAF group experienced ≥ 7% decline in hip BMD (1.1% vs. 6%) and ≥ 5% decline in spine BMD (11% vs. 25%) in comparison to patients in the TDF group [43]. Biomarkers associated with bone resorption and formation showed significantly smaller changes from baseline at weeks 48 and 96 in patients treated with TAF vs. TDF in both the studies [43, 57, 58].

#### Renal safety (up to 144 weeks)

A significantly smaller reduction in eGFR in the TAF vs. TDF groups was noted at week 48, in both the studies (median change in estimated GFR: study 108: − 1.8 mL/min vs. − 4.8 mL/min; *p* = 0.004; study 110: − 0.6 mL/min vs. − 5.4 mL/min; *p* < 0.0001) [57, 58]. Renal safety with TAF was sustained at week 96 (change in eGFR from baseline in the pooled analysis: − 1.2 mL/min vs. − 4.8 mL/min in the TAF vs. TDF groups, respectively; *p* < 0.001) [43], and week 144 (change in eGFR from baseline in the pooled analysis at week 144 in the double-blind extension phase: − 1.2 mL/min vs. − 6.0 mL/min in the TAF vs. TDF groups, respectively; *p* < 0.001) [59]. In the real-world study by Kaneko et al*.*, while both TAF and TDF were well tolerated, a significant decline in estimated glomerular filtration rate (eGFR) was noted only in the TDF group at week 48 [62].

### Switch-over from TDF to TAF

The one-year results of the open-label extension phase of studies 108 and 110 revealed maintenance of viral suppression, with significantly higher normalization of ALT levels with 144 weeks of TAF therapy [67, 68]. A significant increase was noted in the proportion of patients with normalization of ALT levels after 48 weeks of switch-over (week 144 of TAF therapy), both as per the AASLD and central laboratory criteria [60, 61].

The preliminary results of switch-over from TDF to TAF at 12 and 24 weeks in the open-label extension phase of studies 108 and 110 revealed improved bone and renal parameters with TAF therapy [67]. These results were sustained up to one-year of the open-label extension phase (viz., after 48 weeks of switch over or after up to 144 weeks of TAF therapy). There was an improvement in markers of tubular dysfunction with TAF therapy (overall decrease in serum creatinine: − 0.018 ± 0.064; *p* = 0.008). Further, there was a significant decline in all markers of bone turnover and improvement in hip and spine BMD after 48 weeks of switch-over (hip BMD: + 0.97 ± 2.88, *p* = 0.002; spine BMD: + 2.18 ± 3.36; *p* < 0.001) [60, 65].

A comparison of the safety results between the TDF double-blind extension group (*n* = 211) and TDF to TAF switch-over group (*n* = 180) at week 144 revealed significantly improved bone and renal safety in the latter group. The median changes in eGFR in both the groups were − 0.9 mL/min and + 4.2 mL/min, respectively (*p* < 0.001). The corresponding changes in hip BMD were − 0.02 and + 0.98 (*p* < 0.001) and spine BMD were + 0.26 and + 2.04 (*p* < 0.001), respectively [69].

Considering the high proportion of Asians in studies 108 and 110, these findings suggest that TAF may be an alternative to TDF with better bone and renal safety for the treatment of Asian patients with HBV infection. The outcomes from studies 108 and 110 in favor of better safety with TAF therapy can be further substantiated by the findings from another recent, randomized, double-blind, phase 3, multicenter, non-inferiority study. A total of 490 patients with chronic HBV infection who had received TDF for ≥ 48 weeks were randomized to receive TAF 25 mg or TDF 300 mg once daily. Out of the 243 patients randomized to TAF, 80% (*n* = 195) were Asians and 84% of 245 patients treated with TDF (*n* = 205) were Asians. Forty-eight weeks after switch-over, TAF was found to be non-inferior to TDF for antiviral efficacy (96% in both groups had HBV DNA < 20 IU/mL). The proportion of patients with ALT normalization was numerically high in patients receiving TAF versus TDF therapy. Furthermore, while patients on TAF had a significant increase in BMD at hip and spine from baseline, the TDF group experienced decline in BMD at week 48. The study concluded that TAF, with a comparable efficacy and better safety profile, can be substituted for TDF in patients with chronic HBV infection [70].

Studies in real-world settings in Asia have also assessed the efficacy of switch-over from TDF to TAF in patients with chronic HBV infection. In the study by Kaneko et al*.*, there was no elevation in HBV DNA or HBsAg levels after 24 weeks of switch-over from TDF to TAF. Further, the significant decline in eGFR with TDF was inhibited by switch-over to TAF. The improvement in eGFR with switch-over from TDF to TAF was observed for up to 24 weeks, regardless of the duration of prior TDF therapy. A significant decline was also noted in the urinary β2MG/Cre ratio, a marker of renal tubular disorder, at 12 and 24 weeks of switch-over from TDF to TAF, thus indicating significant improvement in renal function with TDF to TAF switch-over [62].

The improved bone and renal safety with TAF vs. TDF may be substantiated with the findings from a recent single-arm, prospective, non-randomized, crossover, pharmacokinetic study. In this study, switch-over from TDF to TAF resulted in a significant 90% decrease in plasma tenofovir concentrations (TDF: 99.98 ± 2.24 ng/mL vs. TAF: 10.2 ± 1.6 ng/mL; *p* < 0.001), and 2.41-fold increase in cell-associated TFV-DP concentration (TAF: 834.7 ± 2.49 vs. TDF: 346.85 ± 3.75 fmol/10^6^ cells; *p* = 0.004) [71].

While switch-over from TDF to TAF may be associated with an improvement in bone and renal safety in HBV-infected patients, changes in lipid profile have also been noted with TDF to TAF switch-over in people living with HIV. A significant increase in total and low-density lipoprotein cholesterol and decrease in high-density lipoprotein cholesterol has been noted with switch-over from TDF- to TAF-based regimen in HIV-infected individuals [72–74]. However, these results have not been confirmed in HIV-infected individuals with baseline hypercholesterolemia [72]. The clinical relevance of these findings, if any to HBV-infected individuals, remains to be established.

### Switch-Over from ETV to TAF

Few recent studies conducted in Asia have evaluated the efficacy of switch-over from ETV to TAF in chronic HBV-infected individuals. In a study by Uchida et al., in 159 HBV-infected patients treated with ETV followed by TAF, the degree of reduction in serum HBsAg levels was higher during TAF versus ETV administration in patients without cirrhosis and patients with genotype B HBV. About 61% of patients reported feeling satisfied with ETV to TAF switch [75]. In another prospective, single-center, comparative study, switch-over from ETV to TAF was found to be associated with a higher efficacy for reduction in serum HBsAg in patients with low baseline HBsAg level [76].

### Outcomes with TAF in patients with decompensated liver disease and liver transplant recipients

While there are no studies comparing the efficacy and safety of TDF versus TAF in HBV-infected patients with decompensated liver disease, the AASLD guidelines recommend TAF as a safe alternative to TDF in these patients with underlying renal dysfunction and/or bone disease [15]. Studies assessing the efficacy and safety of TDF in chronic hepatitis B-related decompensated cirrhosis also suggest careful monitoring of renal function in these patients [77]. Regarding HBV-infected liver transplant recipients, in a recent retrospective analysis (that included 72% Asians/Pacific Islanders), the use of TAF was associated with less deterioration of renal function when compared to other antiviral agents [78]. In another registry-based study conducted in Asia, switch-over from TDF to TAF prophylaxis in HBV-infected liver transplant recipients was associated with increased normalization of ALT levels and improvement in renal function [79].

## Use of NAs in patients with HBV reactivation

The APASL guidelines recommend initiation of treatment with NAs in patients with HBV reactivation, without delay or waiting for HBV DNA results. The guidelines also recommend considering liver transplantation in patients with severe liver failure and patients with poor prognosis (< 2 log reduction in HBV DNA) two weeks after initiation of NAs [18]. The American guidelines recommend treatment with TAF, TDF, or ETV for treatment of HBV reactivation in HBV/HCV co-infected patients and in patients with HBsAg-positive status after liver transplantation, due to the low rates of resistance with these drugs on long-term use [15]. While, there are no data to support the efficacy of TAF in the focus settings, treatment with TDF has been found to be effective in reducing HBV DNA levels, improving Child-Turcotte Pugh (CTP) and model for end-stage liver disease (MELD) scores and reducing mortality in patients with acute-on-chronic liver failure (ACLF) due to severe HBV reactivation [80]. Further, TDF has been noted to be superior to ETV for treating HBV-ACLF due to HBV reactivation in the short term [81]. A recent open-label randomized controlled study has also suggested addition of telbivudine to TDF to be safe with better improvement in survival versus TDF monotherapy for the treatment of ACLF in cases of spontaneous HBV reactivation [82].

## Indications for the use of TAF in Asia: expert consensus recommendations

Majority of the patients in studies 108 and 110, the pooled analysis of both the studies, and the recent TDF to TAF switch-over study were Asians, thus suggesting TAF as an effective and safe long-term treatment in Asian HBeAg-negative and HBeAg-positive, chronic HBV patients [43, 57, 58]. Further, the presence of risk factors for bone disease, such as age ≥ 50 years, female gender, baseline eGFR < 90 mL/min/1.73 m^2^, or history of osteoporotic fracture, had no influence on efficacy of TAF, both at 48 and 96 weeks.[44, 83]. Fewer patients experienced a BMD decline of > 3% in the TAF vs. TDF groups, independent of the nature and frequency of these risk factors [83].

The expert panel proposed recommendations on the indications for the use of TAF in Asia, after reviewing the available literature and factoring the clinical experiences in Asian settings. These recommendations are listed in Table 4.

**Table 4:** Patients with HBV infection, suitable for treatment with TAF

Patients with HBV infection	Indications for TAF
At risk of bone and renal disease	Age ≥ 50 years
	Patients on hemodialysis
	Patients with eGFR < 60 mL/min/1.73 m^2^ (CKD category 3–5)
	Postmenopausal women
	Obese patients (BMI > 30 kg/m^2^)
With comorbidities at risk of renal disease*	Hypertension
	Type 1 and type 2 diabetes mellitus
	Cardiovascular disease
	Smoking
	Patients at risk of vitamin D deficiency
	Family history of kidney disease
	Lupus
	Multiple myeloma
With comorbidities at risk of bone disease*	History of fractures
	Hyperparathyroidism
	Asthma
	Nutritional or gastrointestinal problems (e.g. Crohn’s or celiac disease)
	Hematological disorders or malignancy
	Hypogonadal states (e.g. Turner syndrome/Klinefelter syndrome, amenorrhea, etc.)
	Endocrine disorders (e.g. Cushing’s syndrome)
	Immobility
Taking drugs inducing renal toxicity*	Anticoagulants, NSAIDs, calcineurin inhibitors
Taking drugs inducing bone toxicity*	Chronic use of steroids, thyroid hormone treatment (l-thyroxine), certain steroid hormones (medroxyprogesterone acetate, luteinizing hormone-releasing hormone agonists), aromatase inhibitors, certain antipsychotics/anticonvulsants/antiepileptic drugs, lithium, methotrexate, antacids, and proton-pump inhibitors
With cirrhosis, or transplantation	
Treated with ETV and planning for switch-over due to resistance issues	

*HBV* hepatitis B virus, *TAF* tenofovir alafenamide, *CKD* chronic kidney disease, *BMI* body mass index, *GFR* glomerular filtration rate, *NSAIDs* nonsteroidal anti-inflammatory drugs, *ETV* entecavir

*The list is indicative and not all-inclusive. It is intended as a guidance/reference

The proposed recommendations are intended to guide the clinicians for the optimization of TAF therapy in patients with chronic HBV infection. The final treatment decision should balance the benefits vs. the risks and the cost of the drug [84]. The reviewed literature clearly highlights the benefits of TAF therapy in terms of both efficacy and safety, along with minimal resistance issues. Improving the access to medication can help further optimize the use of TAF therapy in chronic HBV patients in Asia [22, 24].

## Pearls for clinical practice

Chronic HBV infection has intermediate-to-high endemicity with high associated morbidity and mortality in Asia. Despite the high disease burden, the rate of uptake of HBV antiviral therapy, and the use of effective and safe NA antiviral therapies is very low in the region. Tenofovir alafenamide is a NA with high barrier against HBV resistance and proven long-term efficacy and safety in Asian HBeAg-positive and HBeAg-negative chronic HBV patients. It may be an effective option for ETV-resistant patients and a safer option than TDF. Optimizing the use of TAF and improving vaccination coverage can help scale up the treatment of chronic HBV infection in Asia. The detailed literature review on TAF provided in this article, along with the expert panel recommendations on the indications for use of TAF in Asians, can help guide clinicians in the region on the optimal use of TAF for better outcomes in chronic HBV-infected patients in Asia.
